# Clinical Profile of Patients Admitted With Mucormycosis During the COVID-19 Pandemic in Medicine Emergency of a Tertiary Care Hospital in North India

**DOI:** 10.7759/cureus.29219

**Published:** 2022-09-16

**Authors:** Neeraj Singla, Nalin Sharma, Navneet Sharma, Ashish Behera, Mandip Bhatia

**Affiliations:** 1 Internal Medicine, Post Graduate Institute of Medical Education and Research, Chandigarh, IND

**Keywords:** covid-19, diabetic ketoacidosis, steroids, oxygen therapy, mucormycosis

## Abstract

Background: Severe acute respiratory syndrome coronavirus 2 (SARS-CoV-2), which has taken the world as a storm, has been indisputably found to be associated with mild to life-threatening pneumonia in the majority of patients. Mucormycosis emerged as a life-threatening complication of coronavirus disease 2019 (COVID-19) in India during the second wave of the COVID-19 pandemic. There lies a large lacuna in the understanding of the disease progression and the association of mucormycosis with COVID-19 and the various predisposing factors.

Aim: To assess the pattern, risk factors, and outcome of mucormycosis cases reported to hospitals in North India during the second wave of the COVID-19 pandemic.

Material and methods: An observational, prospective study was conducted for 109 patients reporting to a medical emergency with a history of suspected or confirmed mucormycosis from May 2021 to July 2021. Obtained data were analysed using descriptive statistics and results were expressed as a percentage and mean.

Results: Out of 109 patients, 75 were male and 34 were female with a mean age of 50.6 years, most cases belong to the rural background. The most common types of mucormycosis were rhino-orbital (34.8%), rhino-orbital cerebral (20.18%), and pulmonary (23.8%). The most common risk factors were uncontrolled diabetes (80 %), use of steroids (68.8%), diabetic ketoacidosis (42%), and COVID-19 positive status (66.9%). High mortality of 33.9% was seen in our study.

Conclusions: The most vulnerable group in our study were patients with pulmonary manifestations (51.4%) and patients requiring oxygen therapy (94.6%). Our study found that scoring systems namely the quick sequential organ failure assessment (q SOFA) scoring system (p-value <0.001) along with the Glasgow Coma Scale (p-value <0.038) can be used as a prognostic indicator and good assessment tools for the degree of severity of disease at an early stage.

## Introduction

The coronavirus disease 2019 (COVID-19) infection has taken the world as a storm. It has been indisputably found to be associated with mild to life-threatening pneumonia in the majority of the patients [1]. However, due to certain comorbidities like diabetes mellitus, coronary artery disease, and hospital-acquired infections such as ventilator-associated pneumonia, reports of the development of severe opportunistic infections have been elucidated [2,3]. Mucormycosis developed as a life-threatening complication of COVID-19 in India during the second wave. Even before the pandemic, mucormycosis was evolving rapidly in India among patients with uncontrolled diabetes mellitus [4,5]. A study from India reported a rise in mucormycosis cases from 24.70 cases per year (1990-2007) to 89 cases per year (2013-2015) at a single tertiary care hospital [4]. It is one of the most disastrous complications in uncontrolled diabetics with mortality rates ranging between 40-80%. India contributes to 40% of the global burden of mucormycosis, with an estimated prevalence of 140 cases per million population [6]. Many case series of co-infection of COVID-19 with mucormycosis have been reported [1,7,8]. There lies a large lacuna in the understanding of the disease progression and the co-infection of mucormycosis with COVID-19 and the various predisposing factors. As limited data is available, the current study was undertaken to study the pattern, attributable risks, and the final outcome of mucormycosis in COVID-19 patients.

## Materials and methods

The current prospective observational study was done to find out the risk factors, clinical profile, and outcome of mucormycosis in our region during the second wave of the COVID-19 pandemic. The study was carried out over a period of three months from May 2021 to July 2021 after taking ethical clearance from Institutional Ethical Committee (IEC), Post Graduate Institute of Medical Education and Research (PGIMER), Chandigarh, vide letter no. INT/IEC/2021/SPL-907 dated 07.06.2021.

Patients suspected or confirmed of suffering from mucormycosis who presented to the medicine emergency in our hospital were enrolled in the study. After informed consent to participate in the study, data of the enrolled patients including demographics, acute presentation, chronic diseases, examination and lab investigations, and imaging was collected using the standard Case Record Form (CRF) and were entered in Microsoft Excel spreadsheets. The admission and treatment decisions were taken by the treating physicians as per institutional policy.

For diagnosis of mucormycosis, nasal scrapping was sent to the microbiology laboratory and all samples were examined microscopically by performing potassium hydroxide (KOH) wet mounts. A 20% KOH mount of scraping revealed hyaline broad aseptate hyphae suggestive of a sample positive for the Mucor group of fungi. But as this study was undertaken in medicine emergency, so fungal culture and polymerase chain reaction (PCR) testing could not be done during the COVID-19 pandemic. Non-contrast CT scan of the head was done to rule out cerebral involvement as well as bone involvement.

The patients were followed up after discharge telephonically or physically on weekly basis for four weeks to determine the course and outcome which was recorded in the case record form. The data of various sub-groups of emergency medical patients with co-morbidities were analysed to establish the pattern, attributable risks, and final outcome of mucormycosis during the COVID-19 pandemic.

Categorical variables were reported as counts and percentages. Continuous data were given as mean±SD and range or median and interquartile range. For skewed data, comparison based on the basis of groups (expired/recovered/recovered with morbidity) was made by the Kruskal-Wallis test followed by the Mann-Whitney test for two groups whereas for normally distributed data, ANOVA followed by Post Hoc Multiple Comparisons test were carried out. For skewed data of two groups (COVID-19 positive/negative), comparisons were made by the Mann-Whitneytest. For normally distributed data, a Student t-test was applied to compare two groups. To find independent predictors of mortality; a Logistic Regression Analysis was carried out. A p-value < 0.05 was considered signiﬁcant. Analysis was conducted using IBM SPSS statistics, version 22.0 (IBM Corp., Armonk, NY).

## Results

In the current study, 109 patients were enrolled, 75 were males and 34 were females and among them, 35 patients belong to urban areas and 74 belong to rural areas. Out of which, the quick sequential organ failure assessment (qSOFA) score; was 1 in 41 patients, in 9 patients it was 2, followed by 3 in another 5 patients at the presentation to medicine emergency.

In our hospital settings, oxygenation therapy was used in 80 patients; nasal cannula in 11, non-rebreather mask in 46 patients, venturi-mask in 24 patients. Risk factors and laboratory parameters of patients are illustrated in detail in Table 1.

**Table 1:** Risk factors and laboratory parameters in patients with mucormycosis at the time of admission GCS: Glasgow Coma Scale, P/F ratio: Arterial Oxygen Saturation/Inspired Oxygen, NLR: Neutrophil Lymphocyte Ratio, CRP: C- reactive Protein), A p-value <0.05 is considered significant

	N (%)	Normal Parameters		p-value
		Mean	Std. Deviation	
Age(yrs)	109(100%)	50.697	13.51	0.415
Risk Factors				
Diabetes Mellitus(Duration in yrs)	86(78.89%)	4.889	4.74	0.014
Diabetic Ketoacidosis	46(42.20%)	1.966	2.1950	0.000
Covid positive Status	73(66.97%)			< 0.001
Time interval after which steroids were started after Covid 19 positive report	75(68.80%)	3.973	1.4044	0.003
Pulse	109	96.165	16.3597	0.042
Spo2 (Oxygen saturation by pulse oximetry)	109	91.018	8.0266	0.001
Total GCS (Glasgow Coma Score)	109	13.972	2.6632	0.000
P/F ratio (Pao2/ Fi02)	109	371.743	95.5960	0.000
Hb in gm% (Hemoglobin)	109	11.604	2.1591	0.973
Total Platelet Count(In Lakh)	109	259698.5535	132328.69314	0.746
Total leucocyte count	109	16716.692	12595.7781	0.001
NLR Ratio (Neutrophil-Lymphocyte Ratio)	109	16.335	28.2277	0.000
CRP	109	87.686	71.0770	0.066
Albumin	109	2.9580	.56145	0.603
Urea	109	52.106	41.6510	0.000
Creatinine mg/dl	109	1.2725	1.26248	0.000
Na ( Serum Sodium )	109	135.450	6.9168	0.233
K ( Serum Pottassium)	109	4.0911	.78781	0.721
RBS on admission ( Random Blood sugar)	109	270.284	112.0596	0.301
CT Severity Score (HRCT Chest) High Resolution Computed Tomography	27(24.77%)	15.185	5.1518	0.941
Time Interval after which Mucor mycosis was diagnosed after Covid Positive report	109	8.844	8.9476	0.000

COVID -19 report was positive in 73 subjects before admission and in 44 subjects after admission to the institute. Amongst them, 25 patients with COVID-19 were given remdesivir injections and three patients received tocilizumab injections.

Among the various parameters with respect to outcome, p-value < 0.05 was found significant with respiratory rate >22 (<0.001), q SOFA score (<0.001), patients with pulmonary mucormycosis (<0.001), orbital mucormycosis (0.034), cerebral mucormycosis (0.004) and on steroids (0.049). Diagnosis of pulmonary, orbital, and cerebral mucormycosis was done by computed tomography of the chest, orbits, and brain respectively. Steroids were used in 75 patients prior to admission, out of which 46 patients were on recommended doses defined as 6 mg of dexamethasone, 32 mg of methylprednisolone, 40 mg of prednisolone per day for 10 to 14 days; and 29 patients were started on higher doses which is more than defined recommended dose. The time interval after which steroids were started after the diagnosis of COVID-19 is three days in 24 patients, after five days in 19 patients, after four days in 13 patients, and after two days in 10 patients (Table 2).

**Table 2:** Correlation between various parameters and mortality related to mucormycosis GCS: Glasgow Coma Scale, RR: Respiratory rate, BP: Blood pressure, qSOFA: quick sequential organ failure assessment A p-value <0.05 is considered significant

Parameters	Expired (%)	p value
Total (n=109)	37(33.9%)	
Gender		0.191
Male	25(67.6%)	
Female	12(32.4%)	
Clinical Status		
Altered Mental Status GCS<15	12(32.4%)	0.038
RR>22	29(78.4%)	<0.001
BP<100	5(13.5%)	0.196
Total q SOFA Score		<0.001
0	7(18.9%)	
1	18(48.6%)	
2	9(24.3%)	
3	3(8.1%)	
Types of Mucormycosis		0.027
Rhino-orbital	9(24.3%)	
Rhino-orbital-cerebral	7(18.9%)	
Pulmonary	11(29.7%)	
Rhino-orbital & Pulmonary	8(21.6%)	
Gastro-intestinal Tract	01(2.7%)	
Clinical Manifestations		
Pulmonary Manifestations	19(51.4%)	0.001
Orbital Manifestations	25(67.6%)	0.034
Rhino-sinus manifestations	24(64.9%)	0.073
Cerebral manifestations	7(18.9%)	0.004
Amphotericin		0.108
Conventional	14(37.8%)	
Liposomal	23(62.2%)	
Other Surgical Interventions	25(67.6%)	0.596
Steroids		
Duration of steroids		0.220
<5 Days	9(29%)	
5-10 days	13(41.9%)	
11-14 days	07(22.6%)	
15-28 days	02(6.5%)	
Dosage of steroids		0.821
Recommended Dose	18(58.1%)	
Higher doses	13(41.9%)s	
Aseptate Hyphae	21(56.8%)	0.573
Uncontrolled Sugar	33(89.2%)	0.482
Diabetic Ketoacidosis	15(40.5%)	0.396
Increased Anion Gap	11(35.5%)	0.084
Positive COVID-19 Report	29(78.4%)	0.217
Oxygen Therapy	35(94.6%)	0.002

Fungal aseptate hyphae suggestive of mucormycosis were present in 62 patients and 11 patients with mucormycosis who were without COVID-19 infection. The p-value was found significant in patients with pulmonary mucormycosis (0.048), patients on steroids (0.004), patients who presented with diabetic ketoacidosis (DKA) (0.049), and patients with high anion gap (0.014) (Table 3).

**Table 3:** Comparison of various parameters between COVID-19 positive and negative patients with mucormycosis GCS: Glasgow Coma Scale, RR: Respiratory rate, BP: Blood pressure, qSOFA: quick sequential organ failure assessment, PGIMER: Post Graduate Institute of Medical Education and Research, Chandigarh A p-value <0.05 is considered significant

Parameter	Covid Positive	Covid Negative	p value
Gender:			0.282
Female	29(26.60%)	5(4.5%)	
Male	69(63.30%)	6(5.50%)	
Background:			0.717
Rural	66(60.55%)	8(7.33%)	
Urban	32(29.35%)	3(2.7%)	
Clinical Status			
Altered Mental Status GCS<15	17(15.59%)	4(3.66%)	0.217
RR>22	45(41.28%)	2(I.80%)	0.110
BP<100	7(6.4%)	1(.91%)	0.586
Total q SOFA Score			0.655
0	48(44.03 %)	6(5.50%)	
1	37(33.94%)	4(3.66%)	
2	9(8.2%)	0	
3	4(3.6%)	1(.91%)	
Types of Mucor mycosis			0.713
Rhino-orbital	33(30.27%)	05(4.58%)	
Rhino-orbital-cerebral	18(16.51%)	04(3.66%)	
Pulmonary	25(22.93%)	01 (.91%)	
Rhino-orbital & Pulmonary	13(11.92%)	0	
Orbital	2(1.83%)	0	
Cerebral	2(1.83%)	01(.91%)	
Gastro-intestinal Tract	1(.91%)	0	
Clinical Manifestations			
Pulmonary Manifestations	41(37.61%)	01(.91%)	0.048
Orbital	66(60.55%)	09(8.25%)	0.326
Rhino-sinus manifestations	67(61.46%)	09(8.25%)	0.357
Cerebral manifestations	22(20.18%)	05(4.5%)	0.136
Amphotericin			0.529
Conventional	49(44.95%)	04(3.66%)	
Liposomal	49(44.95%)	07(6.42%)	
Steroids			
Steroids used in patients	72(66.05%)	03(2.75%)	0.004
Steroids Route			1.000
Intravenous	36(33.02%)	01(.91%)	
Oral	36(33.02%)	02(1.80%0	
Uncontrolled Diabetes	82(75.22%)	09(8.25%)	0.875
Diabetic Ketoacidosis	38(34.86%)	08(7.33%)	0.049
Anion Gap			0.014
Increased	23(21.10%)	07(6.42%)	
Normal	57(52.29%)	03(2.75%)	
COVID-19 Positive Status			
Outside Report	73(66.97%)	0	<0 .001
PGIMER Report	44(40.36%)	0	0.014
Oxygen Therapy	78(71.55%)	03(2.75%)	0.001

Outcomes of 109 patients were assessed as 33 were discharged, 39 were discharged with morbidity, and 37 got expired. Morbidity in patients is described as the loss of vision, hemiparesis, cerebral involvement incapacitating patients for activities of daily living, and irreversible lung involvement with a need for domiciliary oxygen support. On applying multivariate regression to find out odd’s ratio, mucormycosis significantly affects patients on oxygenation therapy (p-value =0 .043) with an odd’s ratio (OR) of 5.085 and patients with a high SOFA Score (p-value <0.001) with OR of 3.628. So, mortality was seen more in this subset of patients (Table 4).

**Table 4:** Multivariate logistic regression analysis of mortality risk factors in patients with mucormycosis p-value< 0.05* significant, p-value<0.001** highly significant

Parameters	p value	Odds Ratio	Lower	Upper
Pulmonary Mucormycosis	0.349	1.655	0.577	4.748
Cerebral Mucormycosis	0.648	0.745	0.209	2.646
Total q SOFA Score	<0.001	3.628	1.827	7.203
Oxygen Therapy	0.043*	5.085	1.056	24.493

## Discussion

Mucormycosis is the third cause of invasive fungal infection after Aspergillus and Candida spp in humans [9]. Mucormycosis is a rare opportunistic fungal infection characterized by infarction and necrosis of host tissues due to angio-invasion by fungus. Mucormycosis has been increasingly described in patients with COVID-19 but the epidemiological factors, presentation, diagnostic certainty, and outcome of such patients are not well defined [10].

In our study, more prevalence of mucormycosis was seen in males as compared to females; the reason was unclear, but a study by Restrepo et al. proposed the protective role of oestrogen in females [11]. Gender had no role in the outcome in our study with a p-value of 0.979 which is non-significant similar to a study by Kashkouli et al. where no gender preference was seen in the outcome of patients [12].

Patients from urban backgrounds recovered better and had less morbidity at the time of discharge, though mortality was similar in both urban and rural subsets of the population with a p-value of 0.736. But better recovery in the urban population could be due to advanced and early access to health care facilities, a fact which has not been reported in the literature.

The most common causes attributed to the risk of mucormycosis in a study by Patel et al. and Gupta et al. [13,14] were uncontrolled diabetes (73.7%), irrational use of steroids, long stay in ICU settings requiring prolonged oxygenation therapy in concordance to our study, in which prevalence of diabetes was seen in 86 (78.8%) patients, with uncontrolled levels in more than 90% of patients due to poor compliance of drugs and steroid intake in most of them. Another similar study of 101 mucormycosis patients is by Singh et al. in which 80% of cases had diabetes and 76.3 % received corticosteroids [15].

Diabetic ketoacidosis was found in 46 (42.7%) patients and the anion gap was increased in 30 (27.5%) patients in contrast to a study of 388 patients by Prakash et al. [16] in which DKA was found in 18 % of patients with mucormycosis prior to COVID-19. High glucose levels, low pH on arterial blood gas analysis, free iron, and ketones accompanied with the decreased phagocytic activity of leucocytes, augments the growth of mucor [17].

In the current study, lymphopenia was predominantly seen with a mean NLR (neutrophil-lymphocyte ratio) of 16.33 ± 28.22 with a p-value of 0.000 which is highly significant and consistent with the study by Berlin et al. [18] in which it is highlighted that lymphopenia predisposes the host to the development of a number of opportunistic infections including mucormycosis.

Patients who were on steroids were 75 (68.8%); with a mortality of 41.3% in patients on steroids and 16.2 % not on steroids with a p-value of 0.049 which is significant in concordance with the study by Ribes et al. [19] in which the use of corticosteroids has contributed to patient’s susceptibility to mucormycosis by causing defects in macrophages and neutrophils and/or steroid-induced diabetes. Furthermore, we found that 46 patients were on recommended doses of steroids as per the recovery trial collaboration group [20] and 29 (38.7%) patients were on higher doses of steroids, but the outcomes of patients in both groups were similar with a p-value of 0.821 which was not significant. 

Globally, the case fatality rate for mucormycosis is 46% [21] which is high compared to the current study, it was found to be 33.9% after four weeks of treatment follow-up. In another study by Mehta and Pandey [1], the mortality rate was also higher at 53% with, and 31% without invasive fungal infection in patients with COVID-19.

Most of the patients presented with rhino-orbital involvement (38 ), rhino-orbital cerebral (22), pulmonary involvement only (26), rhino-orbital with pulmonary (13), only cerebral involvement (3), only orbital (2), and one patient each with gastrointestinal (GI) tract, rhino-pulmonary, rhino-cerebral, rhino-cerebral & pulmonary, and rhino-pulmonary involvement and this is consistent with the study by Reid et al. [22] in which primarily six forms of mucormycosis has been described Mortality was 23.7% in rhino-orbital, 31.8 % in rhino-orbital cerebral, and 42.3% in pulmonary, with a p-value of 0.027 which is significant in concordance with a study by Vaughan et al. [23] in which mortality was near 40 % in rhino-orbital cerebral group.

In patients with pulmonary involvement usually manifested with a respiratory rate of more than 22, mortality was 61.7% as compared to 12.9% in patients without tachypnoea with a p-value of <0.001 which is very significant. So, patients with pulmonary mucormycosis either alone or in the association were 42, mortality was 45.2 % and recovery was also similar with a p-value of <0 .001 which is quite significant. In contrast to a study by Pettrikkos et al. [24] and Lin et al. [5] in which mortality was high as 76% and 80% respectively due to misdiagnosis of patients being treated for bacterial pneumonia with high-grade antibiotics for prolonged periods before the final diagnosis is documented in patients with post-COVID-19 who had exacerbating dyspnea which is attributable to uncontrolled blood glucose level and usage of higher doses of steroids as enumerated in the study by Prakash and Chakrabarti [6].

Mortality in patients with cerebral involvement was 25.9% and recovery was also similar (18.5%) with a p-value of <0.004 which was statistically very significant and consistent with the study by Balai et al [25] which highlighted that rhino-cerebral mucormycosis is a life-threatening disease and causes permanent neurological complications.

In patients with a qSOFA score [26,27] of 2 or more, mortality is very high (100%) as compared to patients with score of 1 or less (<50%) with a p-value of <0.001 which is highly significant in concordance with the study done by Valerio Pascua et al. [28] in which qSOFA score of 2 or more is detrimental in predicting mortality in patients with severe COVID-19. In a study by Freund et al. patients with a qSOFA score of 2 or higher had a mortality of 24%, and only 3% for patients with a qSOFA score of less than 2 [27].

Mortality of patients who were on liposomal amphotericin and on conventional amphotericin was 41.1% and 26.4% respectively with a p-value of 0.108, as there was an acute shortage of amphotericin B, especially liposomal during the second wave of the pandemic [29]; so conventional form was used for early treatment basis after strong clinical suspicion but results were not statistically significant in concordance to the study done by Jeong et al. [30], lipid-based amphotericin B formulations did not appear to confer any survival advantage over conventional amphotericin B.

Limitations

Patients referred to a tertiary care centre mostly have the more advanced or refractory disease, and thus may not be demonstrative of the overall patients affected by mucormycosis. Serum iron studies could not be done in patients presenting in emergency settings to ascertain as a risk factor in mucormycosis due to logistic issues. Genus confirmation for mucormycosis, could not be done as most of the patients were handled in emergency settings during the COVID-19 pandemic and fungal culture could not be done.

## Conclusions

In our study, the most vulnerable group were patients with pulmonary manifestations and required oxygen therapy. Patients who had uncontrolled diabetes, diabetic ketoacidosis at the time of admission, COVID-19 positivity, and patients who used steroids were at high risk of developing mucormycosis. At an early stage of admission, the q SOFA scoring system along with Glasgow Coma Scale (GCS) can be used as a prognostic indicator and good assessment tool for the degree of severity of the disease.
